# Tuning Reinforcement Learning Parameters for Cluster Selection to Enhance Evolutionary Algorithms

**DOI:** 10.1021/acsengineeringau.3c00068

**Published:** 2024-04-16

**Authors:** Nathan Villavicencio, Michael N. Groves

**Affiliations:** † Department of Mathematics, California State University Fullerton, Fullerton, California 92834, United States; ‡ Department of Chemistry and Biochemistry, California State University Fullerton, Fullerton, California 92834, United States

**Keywords:** Global Optimisation Search, Agglommerative Clustering, Learning Agent, Evolutionary Algorithm, Material Structure Prediction

## Abstract

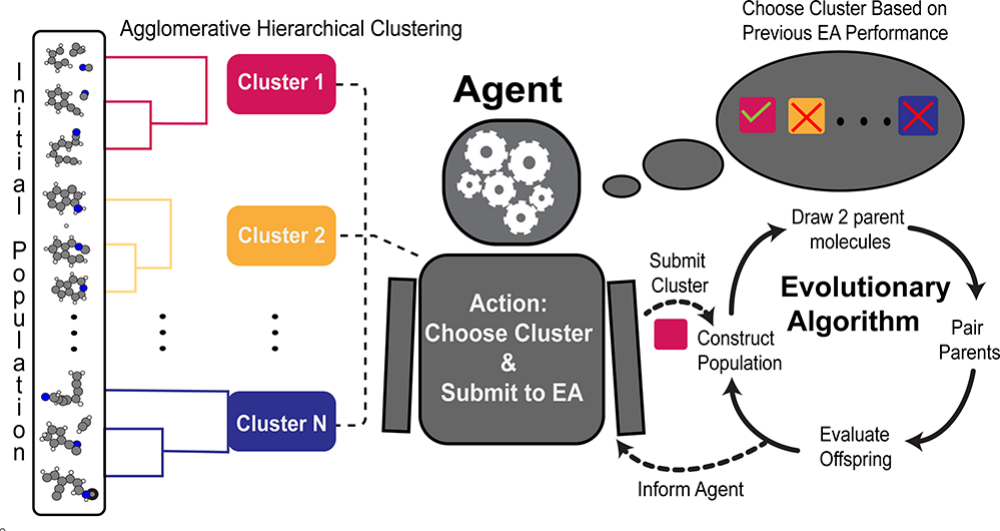

The ability to find optimal molecular structures with desired properties is a popular challenge, with applications in areas such as drug discovery. Genetic algorithms are a common approach to global minima molecular searches due to their ability to search large regions of the energy landscape and decrease computational time via parallelization. In order to decrease the amount of unstable intermediate structures being produced and increase the overall efficiency of an evolutionary algorithm, clustering was introduced in multiple instances. However, there is little literature detailing the effects of differentiating the selection frequencies between clusters. In order to find a balance between exploration and exploitation in our genetic algorithm, we propose a system of clustering the starting population and choosing clusters for an evolutionary algorithm run via a dynamic probability that is dependent on the fitness of molecules generated by each cluster. We define four parameters, MFavOvrAll-A, MFavClus-B, NoNewFavClus-C, and Select-D, that correspond to a reward for producing the best structure overall, a reward for producing the best structure in its own cluster, a penalty for not producing the best structure, and a penalty based on the selection ratio of the cluster, respectively. A reward increases the probability of a cluster’s future selection, while a penalty decreases it. In order to optimize these four parameters, we used a Gaussian distribution to approximate the evolutionary algorithm performance of each cluster and performed a grid search for different parameter combinations. Results show parameter MFavOvrAll-A (rewarding clusters for producing the best structure overall) and parameter Select-D (appearance penalty) have a significantly larger effect than parameters MFavClus-B and NoNewFavClus-C. In order to produce the most successful models, a balance between MFavOvrAll-A and Select-D must be made that reflects the exploitation vs exploration trade-off often seen in reinforcement learning algorithms. Results show that our reinforcement-learning-based method for selecting clusters outperforms an unclustered evolutionary algorithm for quinoline-like structure searches.

## 1 Introduction

When it comes to large molecules, the search for global minima structures is a difficult task, for which many different algorithmic solutions have been proposed. Techniques such as minima-hopping,^1^ Monte Carlo methods,^2,3^ random search,^4^ particle swarm optimization,^5,6^ simulated annealing^7^ via basin-hopping,^2^ neural network based methods,^8^ and evolutionary or genetic algorithms^9−14^ have been implemented to address this search.

An evolutionary algorithm (EA) is a metaheuristic optimization algorithm that is well suited to potential energy surface minimization problems due to their ability to search large regions of the energy landscape and increase the efficiency of the search via parallelization.^15^ The trademark of this algorithm is its creation of a new candidate molecule by the crossover of two parent molecules (randomly selected from a starting population pool) via cut and splice and random changes to the new candidate via mutations.^9,16^ For molecular crystals, symmetric crossover has been used to ensure a parent’s space group is maintained in an offspring structure.^15^ Concepts of Darwinian evolution are used by ranking molecules in the population pool according to fitness and adjusting the probability so that molecules with higher fitness are more likely to be selected.

A shortcoming of a traditional evolutionary algorithm is its tendency to converge on local minima structures rather than global minima. In order to balance exploration and exploitation in an evolutionary algorithm-based search of the energy landscape, techniques such as improving upon the initial candidate pool,^17^ integrating a Bayesian acquisition function into the fitness function,^18^ dynamic management of evolutionary operators,^13^ following the ab initio gradient of the potential energy surface,^14^ and clustering the parent population^11,12,17^ have been proposed.

According to Pereira and Marques, when searching for the global minima potential energy structure (PES), one should consider structural information for estimating dissimilarities between different molecules rather than their fitness values.^19^ Oganov et al. state dissimilar parent structures from different local minima tend to produce offspring with higher energy.^17^ In order to prevent excess computation producing unviable offspring, clustering the parent population by structural similarity has been implemented in multiple works with beneficial results.^11,12,17,20^ In addition to suppressing unfavorable areas of the energy landscape, clustering allows for more exploration by having the evolutionary algorithm search different areas of the energy landscape rather than focusing on the most fit members of the population (exploitation) and possibly converging on local minima.

Previous work has shown that some clusters tend to produce better candidates than others on average.^12^ Therefore, it can be beneficial to spend more time searching for more favorable clusters over others. Sankaranarayanan et al. used clustering and genetic algorithms to create multiple tribes that compete against each other to increase population size. This increase in population size for successful tribes translates to a more expansive search in promising areas of the PES.^21^ In order to search the PES in an efficient but thorough manner, a few different attempts to achieve a delicate balance between exploration of lesser known PES regions and exploitation of regions known to have high fitness have been made.^18,20,21^

In this paper, we choose to address this exploration versus exploitation dilemma by clustering the initial parent population and developing a mechanism to rationally select these clusters for subsequent evolutionary algorithm runs based on their performance. By doing this, we equate the problem of finding global minima structures to the multiarmed bandit problem. The multiarmed bandit problem is a problem in which a decision maker iteratively selects from a fixed set of choices where the properties of each choice are only partially known. The multiarmed bandit problem stands as a classic challenge in reinforcement learning, illustrating the dilemma of balancing exploration and exploitation. Reinforcement learning is considered one of the three basic machine learning paradigms alongside supervised and unsupervised learning methods. In reinforcement learning, an agent learns which actions are most beneficial through a process of trial and error; after each action, the rewards/consequences of this action are calculated by a defined reward function, and a policy on how to effectively use these actions are developed. Because of the ability of reinforcement learning to address the multiarmed bandit problem, we decided to implement it as a solution to global minima searches via evolutionary algorithms with clustered populations.

We offer a novel approach to this problem of balancing exploration versus exploitation by using a reinforcement learning algorithm to select the clusters used in evolutionary algorithm runs. In our self-developed reinforcement learning algorithm, we define four learning parameters (MFavOvrAll-A, MFavClus-B, NoNewFavClus-C, and Select-D) that increase or decrease a cluster’s likelihood of subsequent selections for an evolutionary algorithm run based on its previous performance. MFavOvrAll-A, MFavClus-B, NoNewFavClus-C, and Select-D correspond to a selection probability increase for producing the most favorable structure overall, increase for producing the most favorable structure from its own cluster, decrease for not producing a most favorable structure, and decrease for overselected clusters, respectively. Our results show that the best parameter combination requires a fine balance between parameter MFavOvrAll-A and parameter Select-D that mirrors the need to balance exploration and exploitation in searches.

## 2 Methodology

### 2.1 Computational Details

The evolutionary algorithm used in this research is implemented using the Atomic Simulation Environment (ASE).^22,23^ The EA produces new molecules by picking two parent molecules from the population and combining them with the cut-and-splice operator.^9,16^ Mutations are not used here given that they tend to enhance exploration, and we want to test the exclusive effect of the learning agent. The initial parent population and all subsequent offspring are locally optimized using density functional tight binding (DFTB) theory via the DFTB+ program package using the parameter set “matsci-0-3”.^24−26^ The accuracy loss resulting from the utilization of DFTB is not a significant concern in our case, as our primary emphasis is on the EA’s capacity to produce energetically favorable structures rather than precisely determining their energy levels.

### 2.2 Data Set

In this paper, we will focus on structures with the chemical composition C_9_H_7_N in order to test how effective our dynamic cluster selection based evolutionary algorithm is. Our version of DFTB+ identifies 4h-quinolizin-4-ylidene as the lowest energy structure and quinoline as the second lowest energy structure. We therefore tasked our algorithm with finding either molecule. We chose this system to test our method because previous research has found this problem to be sufficiently challenging to easily gauge the success of different method implementations.^11,12^

The library of structures that were used for the initial pool of candidates was generated following an in-house algorithm that builds molecular structures atom-by-atom. When a certain stoichiometry is assigned to this locally developed code, it randomly picks one of the atoms from the list of possible choices. Depending on the element, one of the possible hybridization geometries to that element is assigned to the atom. For example, if a carbon atom is chosen, it can be assigned either sp, sp^2^, or sp^3^ hybridization. This defines where the next randomly selected atom is placed at a distance equal to the covalent radii of both atoms added together. As each new atom is placed and the geometry of its connections to future atoms assigned, the code also checks if the atom is too close to any other atoms, defined as less than 70% of a bond radius, as determined above. If two atoms are too close, then the process starts over. The code was executed using two strategies for hybridization assignment: equal likelihood of all hybridizations, and predetermined nonequal assignments. In the equal likelihood regime, the hybridization is assigned randomly with equal probability for all types. For the predetermined nonequal assignment regime, the total probability of selecting any hybridization is 100% but it is not equally distributed. Instead, varying probabilities for different assignments were run. For example, in one run, one hybridization had a 100% probability of being applied each time, while the others have 0%. In another run, one hybridization would have a 75% chance of being selected, while the remaining 25% is distributed among the other options. This process produced a large variety of chemically relevant structures and is summarized in a paper by Kellas and Groves.^11^ A similar strategy is implemented for molecular crystals in the Genarris code.^27^ Using the Kellas and Groves method, we generated 812 molecules in the ASE trajectory format.^22,23^

### 2.3 Clustering

In order to cluster molecules, we employ a fingerprinting function to measure their dissimilarity. There are many fingerprinting functions to choose from including atom-pairs,^28^ bag-of-bonds,^29^ atom-centered symmetry functions,^30^ smooth overlap of atomic positions,^31^ overlap matrix fingerprint,^32^ and the partial radial distribution function.^33^ Many of these examples seem to follow a similar idea of quantifying the local environment where some form of a two-body term is used, while the more involved ones also try to quantify three- and four-body terms. We intend to extend this work to larger structures; however, to test the learning agent, we are going to use a well-established fingerprint function that focuses on quantifying two-body terms. Following the work of Oganov et. al.,^34^ we implement the following fingerprint function
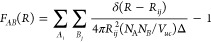


**Figure 1:**
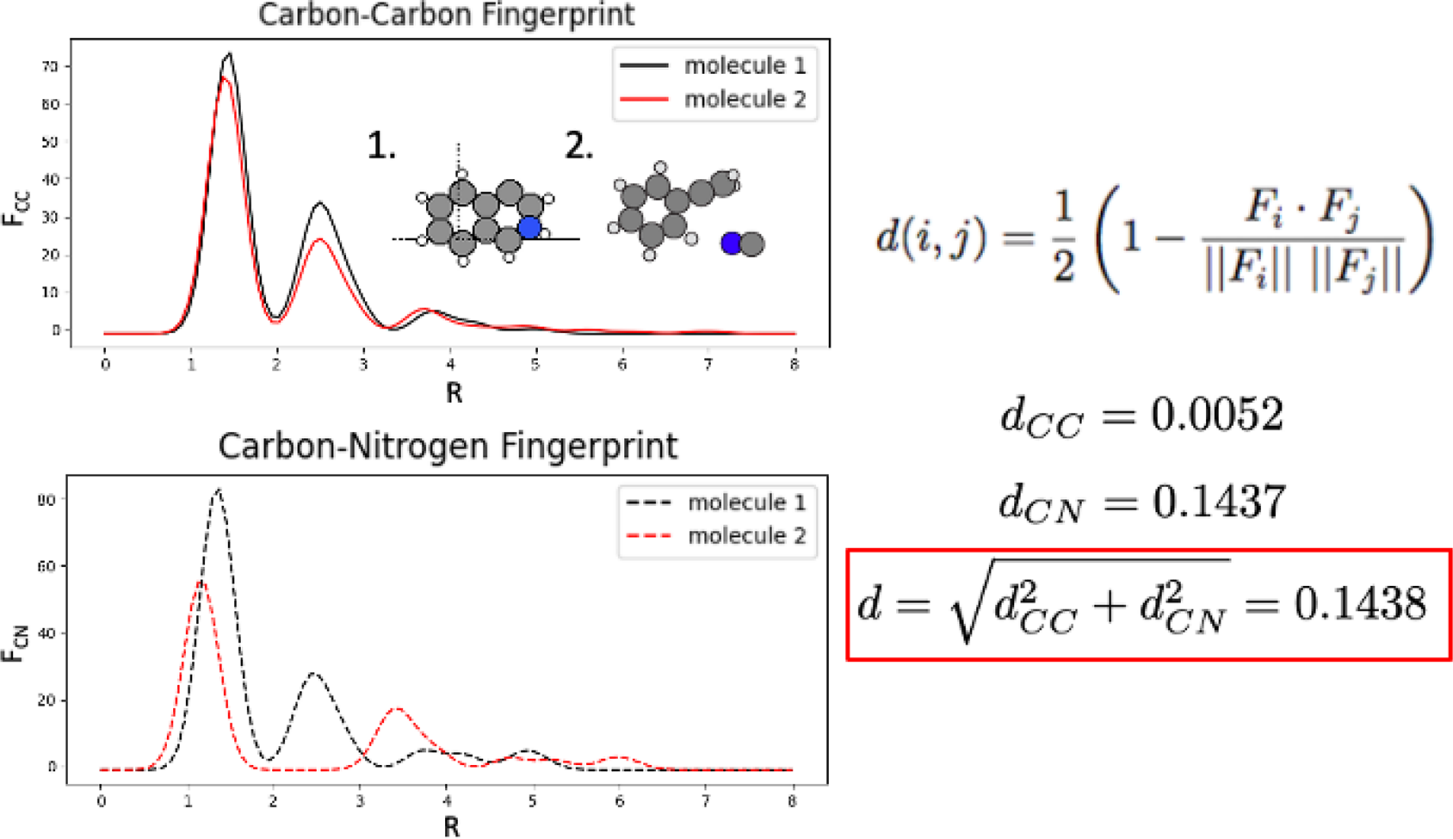
Left: The carbon–carbon fingerprint (top) and carbon–nitrogen fingerprint (bottom) of two molecules. Right: The distance between the two molecules is then generated using the cosine distance function on both fingerprints.

This fingerprint is a radial distribution function where *i* = 1, 2, ..., *N*_*A*_, *j* = 1, 2, ..., *N*_*B*_, *R*_*ij*_ is the distance between atom *A*_*i*_ and atom *B*_*j*_, and *V*_*uc*_ is the unit cell volume. Each peak is smoothed before calculating the sum using a Gaussian kernel, δ, and accumulated into a histogram with bin size Δ; we use the values δ = 0.2 and Δ = 0.5 Å. Because we are not dealing with a molecular crystal, the unit cell volume is purposeless in our calculation and acts more as a scaling factor. We set *R*_*max*_ = 8 Å and . We set *R*_*max*_ to 8 Å because most molecules had interatomic distances below this value, allowing us to consider the contributions of all carbon–carbon and carbon–nitrogen distances (for feasible molecules) while preventing too much of the fingerprint from extending too far and diluting our distance metric. Since we are dealing with quinoline (C_9_H_7_N), we only utilize the carbon–carbon fingerprint, *F*_*C*,*C*_, and the carbon–nitrogen fingerprint, *F*_*C*,*N*_. Following similar research,^12^ we assume any contributions from the hydrogen atoms are redundant and do not include them in our calculation. The fingerprints of the two molecules can be seen in Figure 1.

Once the fingerprint of each atom has been calculated, we calculated the distance between each molecule using the cosine distance function. When comparing two molecules, we produce two distances: the first between the carbon–carbon fingerprints and the second between the carbon–nitrogen fingerprints. We then calculate the total distance using the distance formula (see Figure 1). We allow the carbon–carbon and carbon–nitrogen distances to contribute equally to the total distance and though we recognize it is possible to prioritize one distance over another with the inclusion of a scaling factor or coefficient, we choose not to do so here.
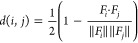


Once the distances between all 812 generated molecules are calculated, we create a distance matrix and use agglomerative clustering via the scikit-learn aggolomerative clustering library to place these 812 molecules into clusters. Agglomerative clustering is a hierarchical clustering technique in which each molecule starts in its own cluster and clusters are merged according to the defined linkage setting. Scikit-learn has four possible linkage settings:

1. **Ward:** minimizes the variance of the clusters being merged.
2. **Average:** uses the average of the distances of each observation of the two sets.
3. **Complete or maximum:** uses the maximum distances between all observations of the two sets.
4. **Single:** uses the minimum of the distances between all observations of the two sets.

**Figure 2:**
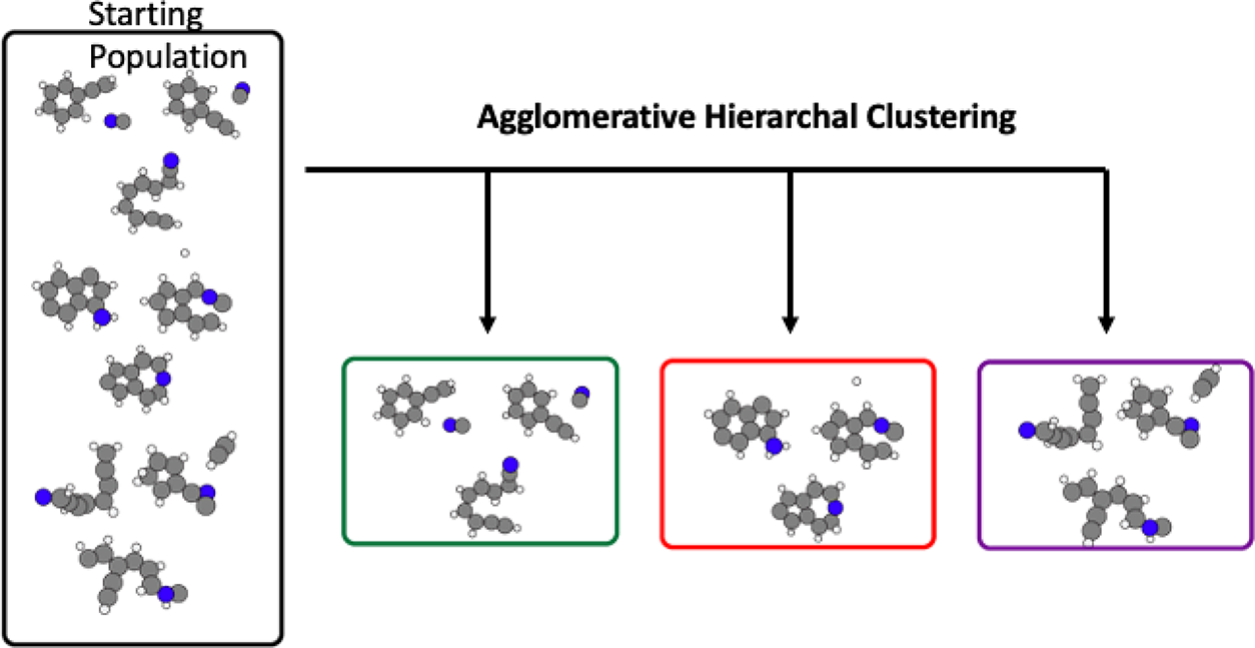
Extremely simplified version of our clustering model’s performance on a small set of molecules.

The goal for clustering was to produce clusters of similar sizes with no cluster having fewer than 20 molecules. We found that only the “complete” linkage setting managed to prevent the creation of a single, large, and central cluster and significantly smaller outlier clusters. After choosing this “complete” linkage setting, a threshold value of 0.026 was picked through trial and error. This means that the distance between the two most dissimilar molecules is calculated and used to determine whether a cluster should combine. If that distance is above 0.026, the clusters will not combine because of our set threshold. The threshold value of 0.026 is likely specific to our quinoline-like structures and was found to produce a notable number of clusters of similar size. Figure 2 shows an example of two representative structures from three clusters.

**Figure 3:**
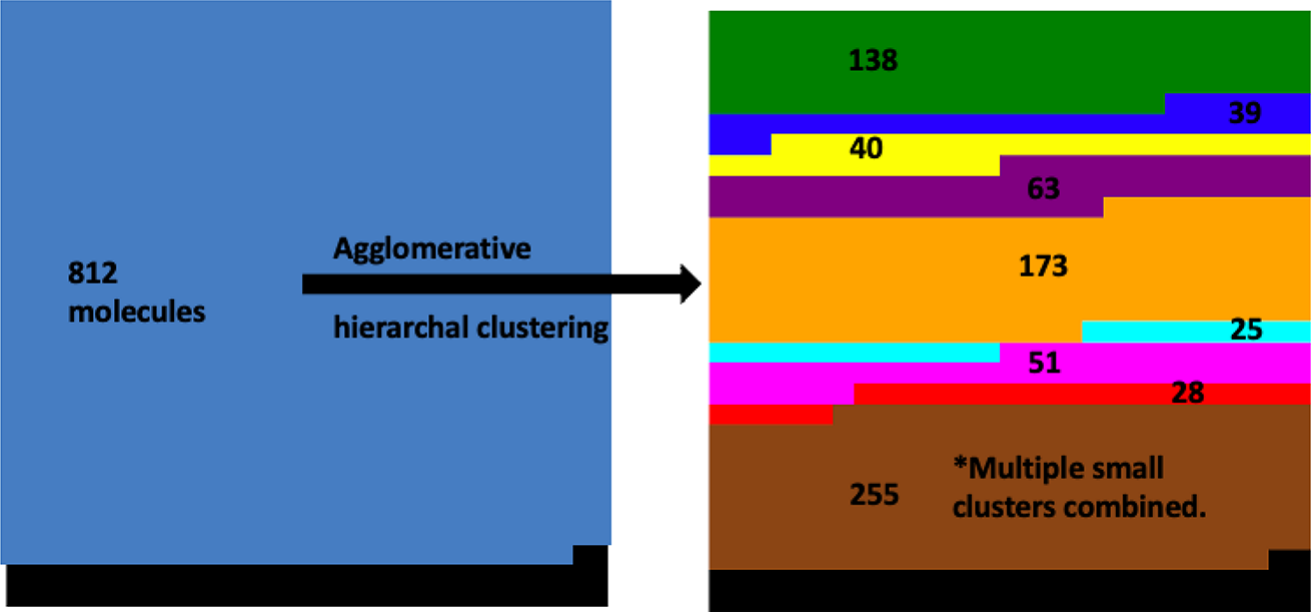
Illustration of how the 812 molecules were distributed among each of the 9 clusters. The final cluster (brown) is consists of many smaller clusters that had to be manually combined.

Molecules that were too dissimilar to be put into clusters of size *n* ≥ 20 were all put into one cluster. Using this method, we clustered 812 molecules (generated by the variational hybridization method outlined earlier in the methodology) into 9 clusters, making sure the global minima structure was not already included. An extremely simplified version of this can be seen in Figure 3. The number of clusters being set to 9 was not an intentional choice but rather reflects a need to balance the formation of a large central cluster and having too many small groups. If the distance threshold is set too high, one large cluster forms, with many small clusters (*n* ≤ 5). If the distance threshold is set too low, all except one or two clusters have less than 20 molecules. Even with our carefully tuned distance threshold, we still needed to create a new “misfit” cluster (brown in Figure 3) comprised of clusters too small to use in our EA algorithms.

### 2.4 Tuning Learning Parameters

In order to determine how often a cluster should be chosen, each cluster is assigned a probability that changes, depending on its performance throughout the evolutionary algorithm run. In order to select a cluster, we use the random.choices() method from the Python random module where we define the weights that dictate how likely a cluster is to be chosen. For example, if molecules 1, 2, 3, and 4 had relative weights of 10, 20, 15, and 5, this would correspond to selection likelihoods of 20%, 40%, 30%, and 10%, respectively. Each cluster starts with its weight set at 100 and is then modified by our reinforcement learning system defined below. The choice to set these weights at 100 was somewhat arbitrary. We wanted the weights to be large enough that we could leave our learning parameters (defined below) as integer values.

**Figure 4:**
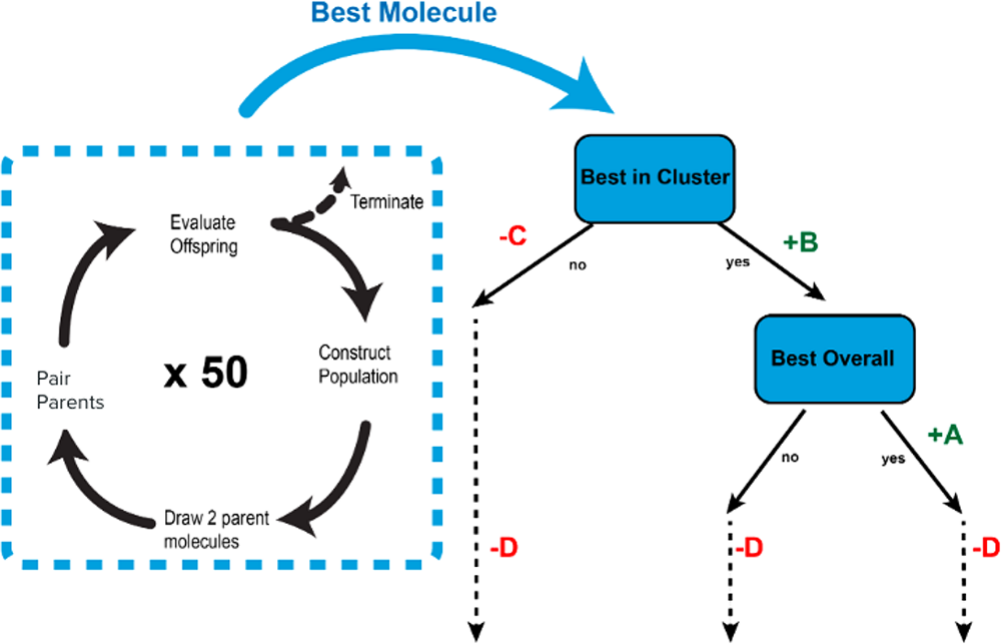
Diagram outlining the how the selection weight of a cluster changes based on its performance with our defined parameters. Each cluster begins with a selection weight of 100.

We defined a set of rules with variable parameters that attempt to find the optimal balance between exploration and exploitation by changing the cluster selection weights throughout the evolutionary algorithm run. The incorporation of these variable parameters MFavOvrAll-A, MFavClus-B, NoNewFavClus-C, and Select-D are detailed below and illustrated in Figure 4.

1. If a chosen cluster produces the lowest energy molecule of any cluster, the relative probability of that cluster being chosen in the future is increased by MFavOvrAll-A.
2. If a chosen cluster produces the lowest energy molecule in its own cluster, the relative probability of that cluster being chosen in the future is increased by MFavClus-B.
3. If a chosen cluster does not satisfy condition 2, its relative probability is decreased by NoNewFavClus-C, where a cutoff exists to prevent negative probabilities.
4. The chosen cluster will have its relative probability decreased by the ratio of how many times the cluster has been selected multiplied by Select-D.

Parameters MFavOvrAll-A, MFavClus-B, and NoNewFavClus-C all work to prioritize high performing clusters by increasing or decreasing a cluster’s selection weight by a constant amount. The intention of Select-D is to prevent a successful cluster from dominating the selection cycle and to promote the selection of clusters that have shown subpar performance throughout the dynamic cluster selection based evolutionary algorithm process. The rationale is that every cluster is capable of producing the lowest energy molecular structure, so we want to give each cluster a chance while spending more time on clusters with favorable outputs.

Unlike MFavOvrAll-A, MFavClus-B, and NoNewFavClus-C, Select-D does not decrease a cluster selection weight by a constant amount. Instead we use a ratio which allows us to more effectively promote exploration since an overselected cluster is receiving a harsher penalty than its underselected counterpart would. Also, if a cluster had a poor performance initially but began producing molecules of interest later in the process, the rise to a higher selection probability would be easier.

Optimizing the variable parameters via evolutionary algorithm runs is extremely computationally expensive. In order to reduce computational time and resources, we modeled the performance of each cluster as the initial parent population of an evolutionary algorithm by taking the mean and standard deviation of molecular energies produced by 100 evolutionary algorithm runs. Modeling the values produced by each cluster as a Gaussian distribution, we can then quickly produce values from each cluster and calculate the cumulative success of different parameter permutations. In order to find the optimal values of our parameters, we did a grid search of all integer value combinations for MFavOvrAll-A ∈ [0, 80], MFavClus-B ∈ [0, 40], NoNewFavClus-C ∈ [0, 20], and Select-D ∈ [0, 90]. Each grid search began with the integer range [0,20] and was increased by 10 until a decrease in performance was shown.

**Figure 5:**
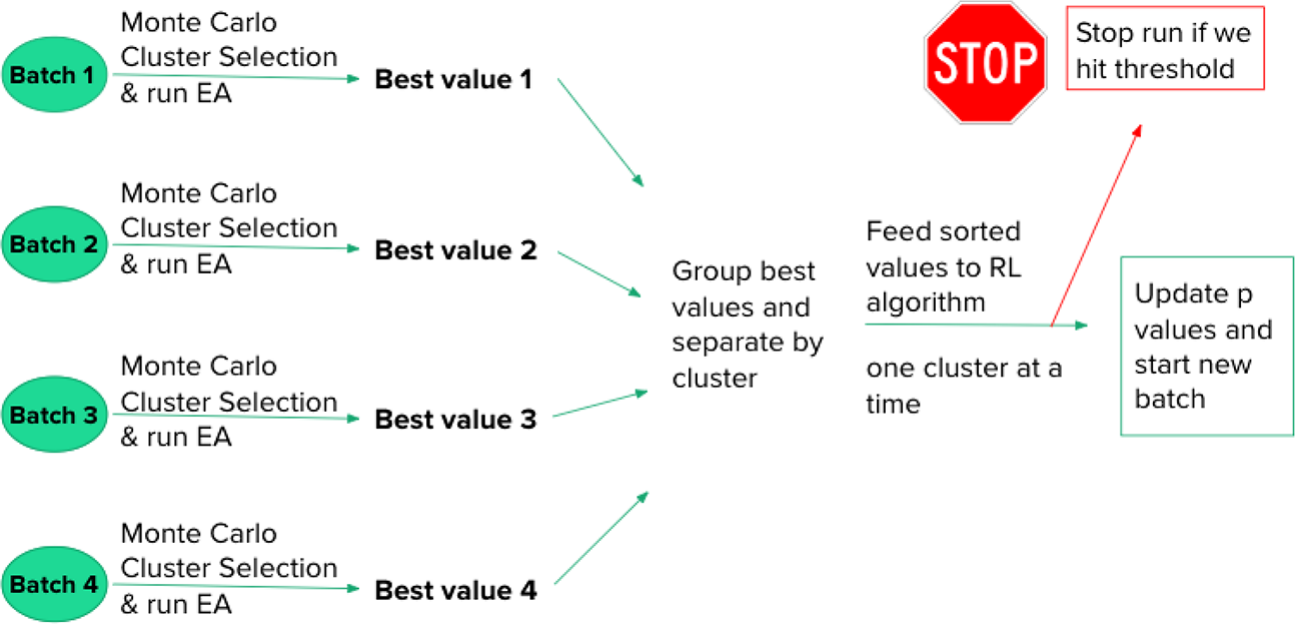
Diagram outlining each of the 100 simulated EA trials used to test a learning parameter combination’s performance. Batches are used here to simulate parallelizing each EA trial.

As stated above, each parameter combination will be used for 100 evolutionary algorithm trials (modeled by using a Gaussian distribution) and have its performance evaluated by the cumulative success. Specifically for the task of finding the optimal parameter combination, we segment each EA trial into batches where 5 clusters are randomly chosen (with replacement so that a cluster can be chosen multiple times), and each chosen cluster simulates an EA run of 50 iterations. Between each batch (selection of 5 more clusters for 50 iteration EA runs), we take the lowest energy values from each cluster and update the selected clusters’ probability for subsequent selection based on our learning parameters. If the an energy value produced is below our defined energy threshold value, *E*_*min*_, the EA trial is stopped and no further batches occur. Each batch consists of 250 Gaussian simulated EA iterations in total, and each trial can run for a maximum of 20 batches. Therefore, if the EA trial runs without producing an energy value below *E*_*min*_ in its 20 batches, the maximum number of iterations (5000) will have occurred. This process is illustrated in Figure 5.

The segmentation of each of the 100 trials per parameter combination into batches was meant to simulate parallelization of each actual EA run with the optimized learning parameters. Ultimately, we found it more suitable to parallelize the actual EA trials without batches (one 50 iteration run at a time). This means each EA trial takes longer, but we can run more of them at a given time. The effects of the batches being used in the testing of the learning parameters but not the actual EA runs will be discussed further in the Discussion section.

**Figure 6:**
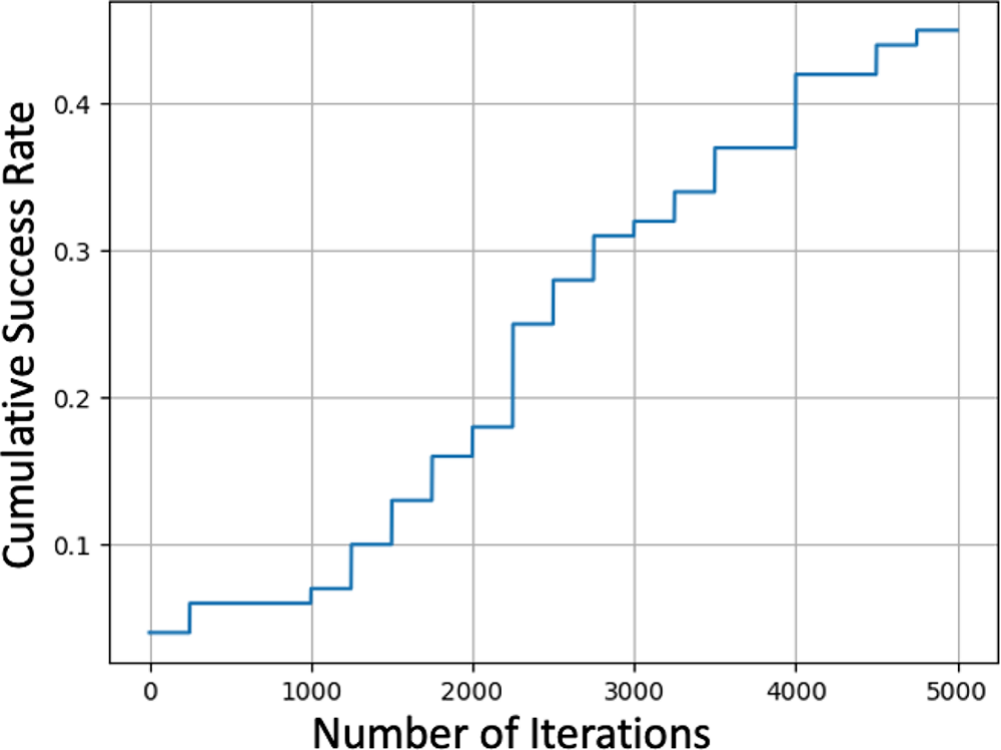
Example of a cumulative success rate generated during each parameter simulation. In this cumulative success rate plot, a = 79, b = 3, c = 19, and d = 68.

In order to determine the performance of each parameter combination, we ran 100 simulation trials for each combination and generated a cumulative success graph. The performance of each parameter combination was then compared by calculating the area under the cumulative success graph. An example of a cumulative success graph is shown in Figure 6.

Each parameter combination MFavOvrAll-A, MFavClus-B, NoNewFavClus-C, and Select-D had 100 trials performed in order to create a metric related to its average performance. Each of the 100 trials consists of up to 5000 iterations. In each trial, a success is defined as producing an energy value below a defined value *E*_*min*_ (energy of quinoline as defined by DFTB+ in this paper). Each iteration produces a lowest energy value; if that lowest energy is below *E*_*min*_, then that trial is considered a success, the trial is stopped, and the iteration number is noted.

The cumulative success graph then measures how many successes have been recorded in X iterations or less. The cumulative success graph starts at zero and by the 5000 iterations could go up as far as 1. However, if any of the trials fail to produce an energy value below *E*_*min*_ in 5000 iterations, then the trial never succeeds and the final cumulative success value (at 5000 iterations here) is less than 1. We purposefully set *E*_*min*_ at a value where no trial is expected to reach 1 by 5000. This makes it easier to differentiate between the top parameter combinations and is a major motivation behind selecting quinoline-like structures.

Since the graph is cumulative, the earlier a parameter combination’s trials begin to mark success, the higher the area underneath a cumulative success graph (cumulative success area) will be. While we think taking the average number of iterations needed for success could be sufficient as well, we believed the cumulative success area would more accurately characterize a parameter combination’s success.

Of the 5,760,000 combinations attempted, the highest performing parameter combination is taken, and the dynamic probability-based cluster selection EA was implemented.

### 2.5 Evolutionary Algorithm Implementation

The EA used in this work is the implementation included in ASE^22,23^ as described in a paper by Vilhelmsen and Hammer.^10^ A population can be generated randomly from a predefined set of structures or a combination of both. Each member of the population is assigned a fitness which is quantified in eq 1:
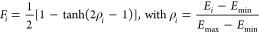
1where *E*_min_ and *E*_max_ are the minimum and maximum energy of any structure in the population. To ensure that a diverse set of candidates can be selected, the probability of selecting a given candidate also relies on a uniqueness factor that tends toward zero the more times the candidate is selected and based on the number of structurally equivalent structures outside of the population. The functional form of the uniqueness factor is quantified in eq 2:
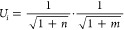
2where *n*_*i*_ is the number of times candidate *i* has participated in a pairing and *m*_*i*_ is the number of candidates which are structurally similar to the *i*th candidate but not included in the population because the *i*th candidate is already present. The probability that a given candidate is selected is *P*_*i*_ = *F*_*i*_·*U*_*i*_. Vilhelmsen and Hammer^10^ find that the inclusion of the uniqueness factor increases the effectiveness of the EA.

The cut-and-splice method used to create candidate structures once two parents are selected was devised by Deavon and Ho.^9,35^ In short, the process involves creating a dividing surface and orienting it randomly through the two parent structures’ center of mass. This cuts the two parent structures roughly in half. The newly formed candidate structure is then composed of the atoms from one parent structure on one side of the dividing surface and the atoms from the second parent structure on the other side of the dividing surface. If the stoichiometry of the system is not maintained, then the following rules are applied: if there are too many atoms of a certain element, then the extra atoms that are furthest away from the dividing plane are removed. If there are too few atoms of a certain element, then atoms are randomly added from the parent structures that were not originally included in the new candidate structure. Finally a check is performed to ensure that atoms are not too close together. If any distance is smaller than the set threshold, the new candidate is rejected, and the process starts again with a new randomly oriented dividing plane. Once a candidate is created, the resulting structure is relaxed 100 SCF cycles or to a maximum force of 0.05 eV/Å with the DFTB+ calculator. This is to limit resources being tied up in trying to fully relax a structure that is not viable. Once one of these two criteria is met, the relaxed structure is compared to the weakest member of the current population. If it is more favorable, then it is added at the expense of the weakest member. If it is not more favorable than the weakest member, then it is rejected. In either case, this process of selecting two parents, creating a candidate from them, relaxing it, and comparing it to the members of the current population counts as a single iteration of the EA.

For the evolutionary algorithm implementation, we will be testing the performance of an unclustered EA and three different versions of a dynamic probability based cluster selection EA where the method for the formation of the parent population varies. We will now outline the differences in methodology between each version and the measures taken to produce comparable results.

The unclustered evolutionary algorithm represents a baseline EA that we can use to analyze the performance of our dynamic probability-based cluster selection method. In the unclustered EA, the entire initial population of 812 molecules is drawn from parent molecules. The molecules produced during each step of the EA run are added to the parent population and can be drawn from in the future. For all EA methods, molecules with lower energies are deemed more fit and are more likely to be chosen as the parents. Each unclustered EA trial consists of 5000 iteration steps, where each step corresponds to the selection of two parents, production of a child molecule via cut-and-splice, and evaluation of the child molecule. 500 EA trials were completed in total to estimate the cumulative success of this method.

For the dynamic probability-based cluster selection EA, all three versions follow the same implementation, with the exception of the usage of the parent population. In both cluster-selection evolutionary algorithms, 20 molecules from each cluster are randomly selected to form the initial parent population for each cluster. After this initialization, there are up to 100 cluster selections. In each cluster selection, a cluster is chosen to run for 50 iterations steps. This was done to equate the total possible number of iteration steps to the unclustered EA’s 5000 steps. After each cluster selection, each clusters’ probability of selection is updated according to the learning parameters, and the parent population for the next selected cluster is formed.

The difference between the three dynamic probability-based cluster selection EA methods lies solely in the formation of the parent population. All three methods implement an energy threshold, Δ*E*, meant to eliminate redundancy in the parent population and prevent early convergence of local minima in the energy landscape.

In the first version, which we will refer to as method 1, the next parent population is drawn only from the output of the previous EA output from that cluster. In this version, the lowest energy molecules that have no other molecules in its energy threshold are selected. In other words, if the next lowest energy molecule has an energy *E*, and any other molecule (selected or not) has an energy in the range *E* – Δ*E* to *E* + Δ*E*, the molecule will not be selected.

In method 2, the next parent population is also drawn only from the EA output of the previous EA output from that cluster. However, in contrast to method 1, the next lowest energy molecule will only be skipped if the previously selected molecule has an energy between *E* – Δ*E* to *E*. Unlike method 1, molecules that are not selected have no impact for method 2.

In method 3, the next parent population is drawn from the output of every EA output from that cluster. Like method 2, the next lowest energy molecule that does not cross the energy threshold of the previously selected molecule is added to the parent population.

**Figure 7:**
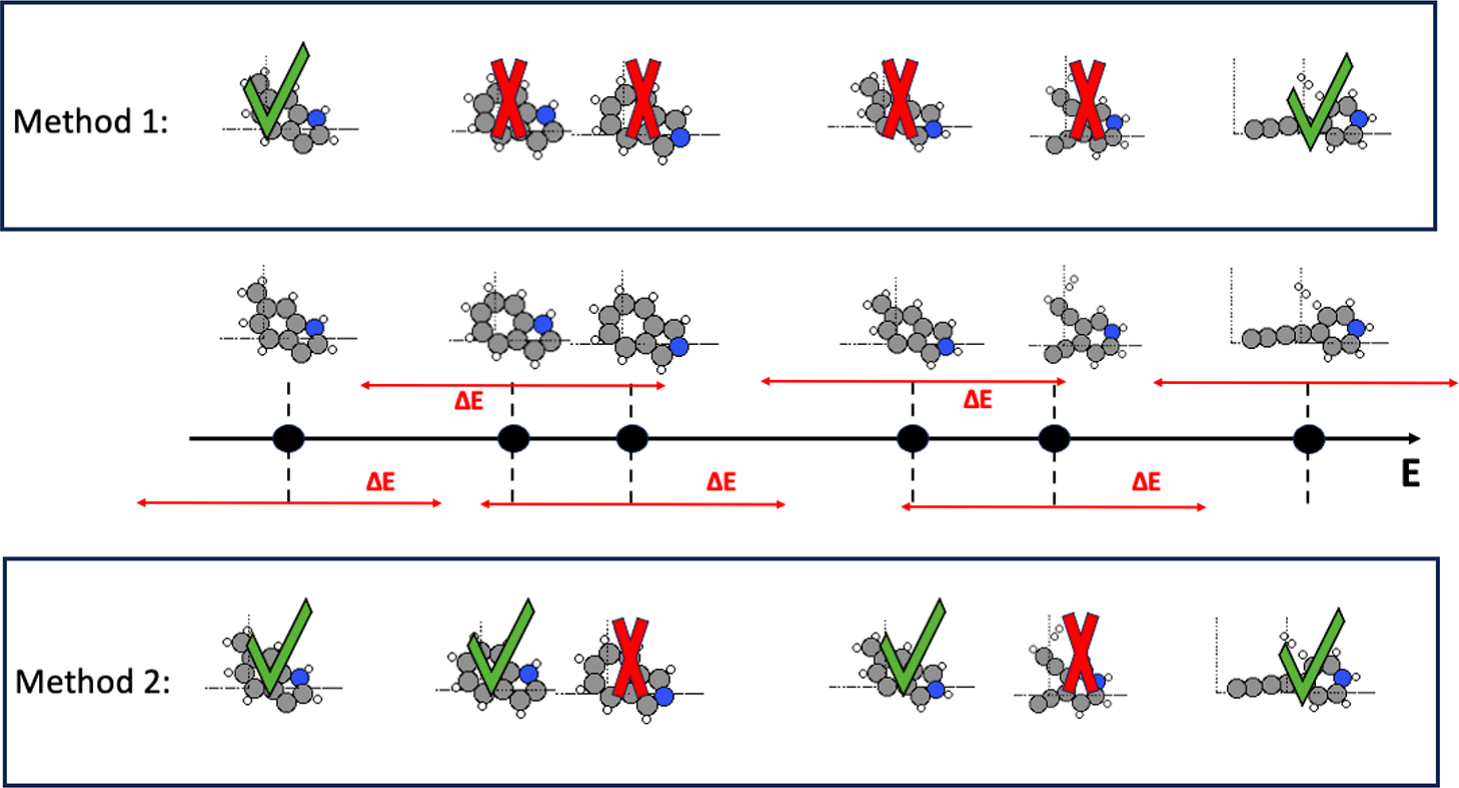
Example of a the parent population selection process for method 1 and method 2. Method 1 will not add a molecule to the parent population if any other molecule within an energy of Δ*E* is present. Method 2 will only reject a molecule if it is within Δ*E* of the previously selected molecule.

**Figure 8:**
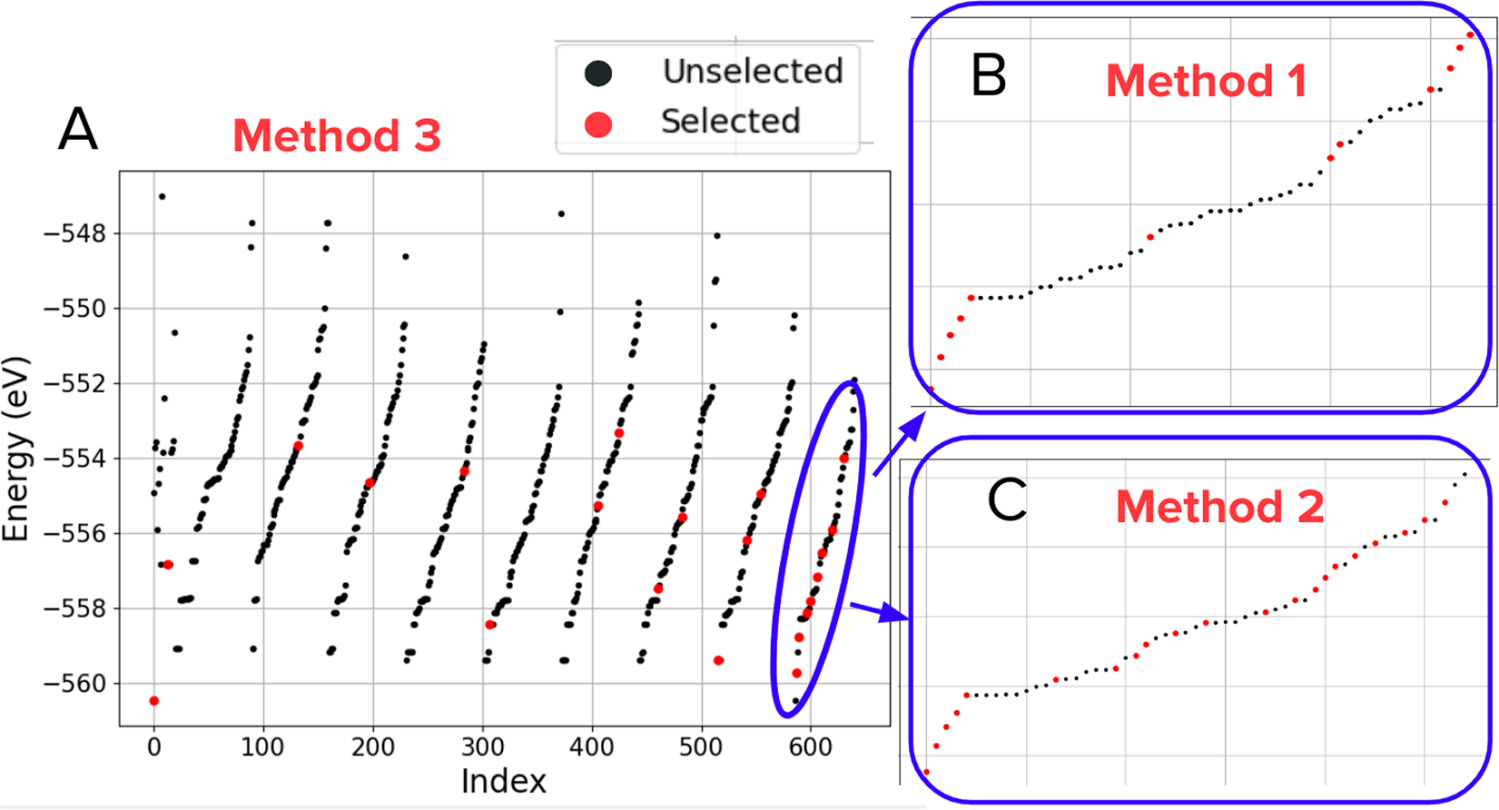
Parent population selection process is outlined for each methods. A: For method 3, all outputs of previous EA runs from that cluster are drawn from with the lowest energy molecule outside of the energy threshold of the previously selected molecule being selected for the next parent population. B: For method 1, only the output of the previous EA run from that cluster is used with molecules being selected for the parent population if no other molecule exists in its energy threshold. C: For method 2, only the output of the previous EA run from that cluster is used with the lowest energy molecule outside of the energy threshold of the previously selected molecule being selected for the next parent population.

The difference between methods 1 and 2 can be seen in Figure 7. Method 3 follows the same selection procedure as method 2 except that it is looking at all molecules previously generated by that cluster’s EA (not just the previous EA iteration). An example of the parent population selection process for methods 1, 2, and 3 can be seen in Figure 8.

Ideally, methods 1 and 2 should be drawing from 70 molecules (50 generated from the previous EA and 20 from the previous parent population). Since method 3 is drawing from all previous outputs belonging to a cluster, it draws from significantly more molecules as the EA run progresses. For Δ*E* values that are too large, it is possible that less than 20 molecules are selected the next parent population. This problem may occur only in method 3 during the beginning phases where each cluster does not have many outputs yet.

The remaining methodologies for these three versions will be identical. These parent population formation methods were employed to allow the EA to continue progressing between cluster selection phases and more accurately reflect the dynamics of the unclustered EA algorithm.

## 3 Results

After clustering the initial 812 molecules^11^ into 9 clusters, 100 evolutionary algorithm runs are done on each cluster with the lowest energy molecule from each run being recorded. The mean and standard deviation of these 100 molecules’ energy are then calculated for each cluster and can be see in Table 1. The mean energy ranged between −558.11 eV and −555.47 eV. On average, cluster 6 produced the lowest energy molecules during its EA runs. When simulating the EA runs, each cluster’s output will be modeled as a Gaussian distribution with the mean and standard deviation calculated in this table.

**Table 1:** Average and Standard Deviation of Lowest Energy Molecules Produced by 100 EA Runs for Each Cluster

Cluster #	1	2	3	4	5	6	7	8	9
Average Energy (eV)	–556.38	–557.24	–555.47	–555.58	–557.54	–558.11	–556.73	–556.78	–557.07
SD (eV)	2.73	2.31	2.59	3.13	1.76	1.79	2.44	2.66	2.48

Performing a grid search of all integer value combinations for *a* ∈ [0, 80], *b* ∈ [0, 40], *c* ∈ [0, 20], and *d* ∈ [0, 90] resulted in 5,760,000 combinations. Table 2 shows the five highest recorded parameter combinations with the highest cumulative success areas, while Table 3 shows the five parameter combinations with the lowest cumulative success areas. From these tables, we can see that the cumulative success areas ranged between 579.5 and 1744.0. The parameters MFavOvrAll-A = 79, MFavClus-B = 3, NoNewFavClus-C = 19, and Select-D = 68 resulted in the largest cumulative success area while the parameters MFavOvrAll-A = 2, MFavClus-B = 28, NoNewFavClus-C = 8, and Select-D = 89 resulted in the smallest cumulative success areas.

**Table 2:** Most Successful Parameter Combinations

MFavOvrAll-A	MFavClus-B	NoNewFavClus-C	Select-D	Success Area
79	3	19	68	1744.0
63	3	4	61	1718.0
73	14	14	64	1710.0
72	22	11	68	1707.0
66	4	10	63	1705.0

**Table 3:** Least Successful Parameter Combinations

MFavOvrAll-A	MFavClus-B	NoNewFavClus-C	Select-D	Success Area
6	20	18	83	593.0
1	21	5	86	590.0
2	31	3	83	583.0
3	10	2	88	582.5
2	28	8	89	579.5

**Figure 9:**
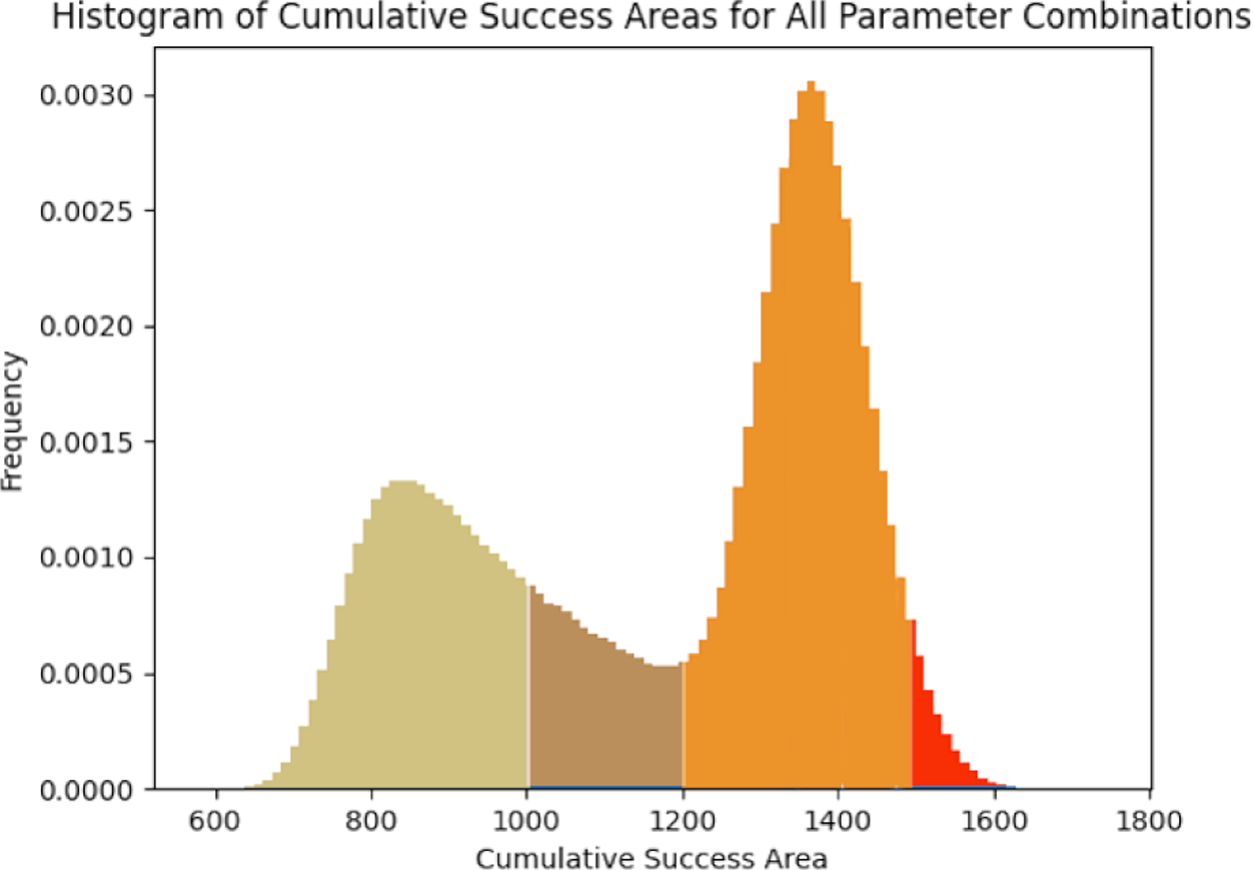
Histogram showing the distribution of cumulative success areas for all parameter combinations. The histogram has been separated into four areas of interest that will be used in later analysis. These areas cover ranges of <1000, 1000–1200, 1200–1500, and >1400.

In Figure 9, all of the cumulative success areas are grouped into a histogram. The histogram is bimodal and has been split into four sections for further analysis. The first section corresponds to cumulative success areas below 1000 and represents the first peak. The second section corresponds to cumulative success areas between 1000 and 1200 and represents the area between the first and second peak. The third area corresponds to cumulative success areas between 1200 and 1500 and represents the majority of the second peak. The last area represents the best cumulative success areas at the right edge of the second peak with a cumulative success area greater than 1500. The second peak is higher suggesting more parameter values are clustered between cumulative success areas between 1300 and 1450. The histogram takes on a bimodal form that we suspect may be a result of most learning parameters either increasing or decreasing the baseline performance (rather than having no effect). However, further work would need to be done in order to validate this suspicion.

**Figure 10:**
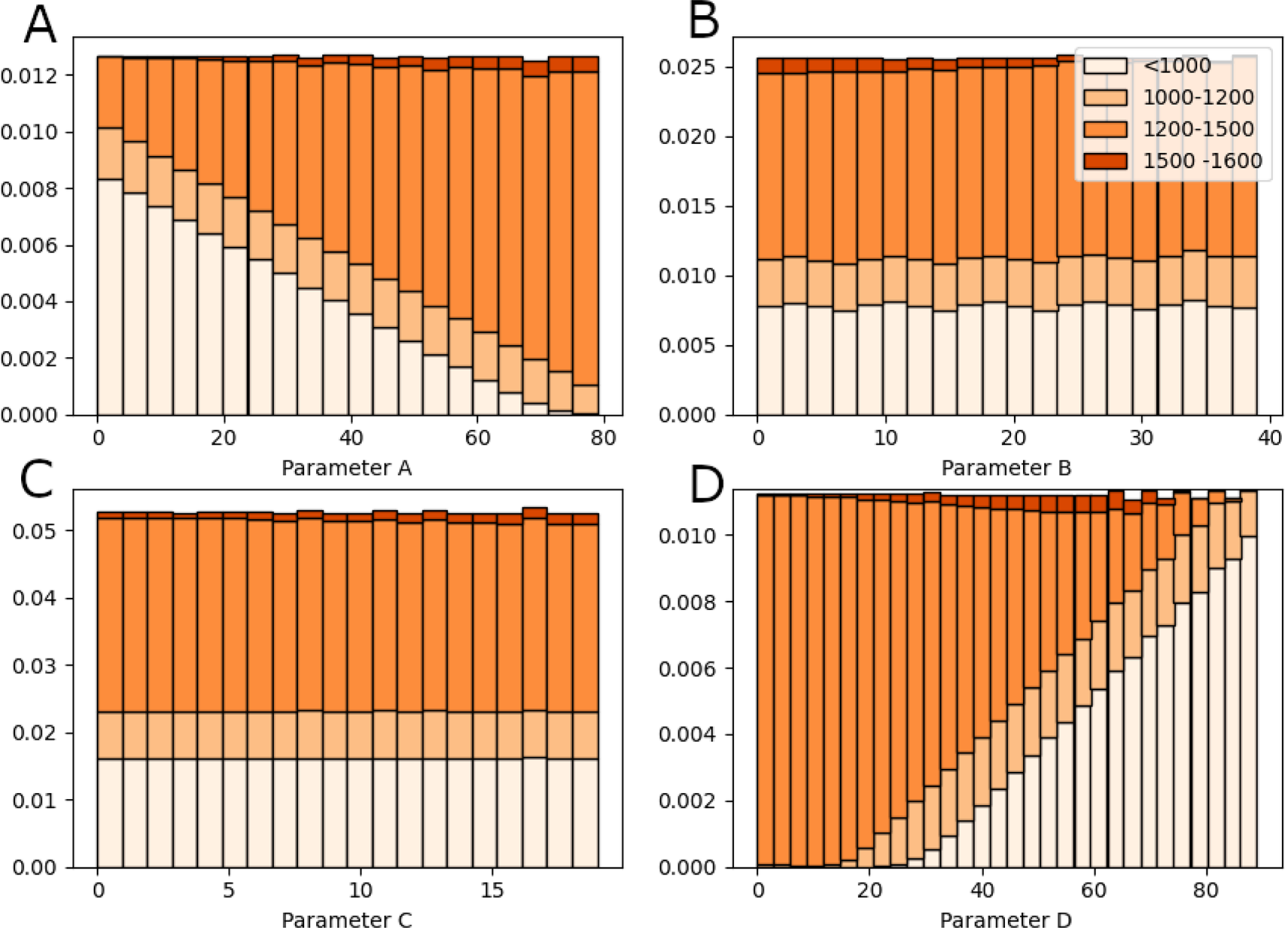
Stacked histogram of each parameter (MFavOvrAll-A upper-left, MFavClus-B upper-right, NoNewFavClus-C lower-left, Select-D lower-right). The cumulative success areas have been split into four areas of interest.

In Figure 10, a histogram of each parameter was then made showing the relationships between parameter values and area on the cumulative success area histogram. The value of parameter MFavOvrAll-A has a clear effect on the value of the cumulative success area. As the value of parameter MFavOvrAll-A increases, the proportion of cumulative success values from the lower value peak increases (see Figure 10A) . Parameter MFavClus-B and NoNewFavClus-C appear to have a much smaller effect on the cumulative success. However, for cumulative success areas between 1500 and 1600, parameter MFavClus-B tends toward smaller values while parameter NoNewFavClus-C tends toward values between toward the upper half of its distribution. (see Figure 10A,B). Upon looking at Figure 10D, parameter select-d appears to prefer values between 40 and 60. This is a consequence of its relationship with A that will be further explored.

**Figure 11:**
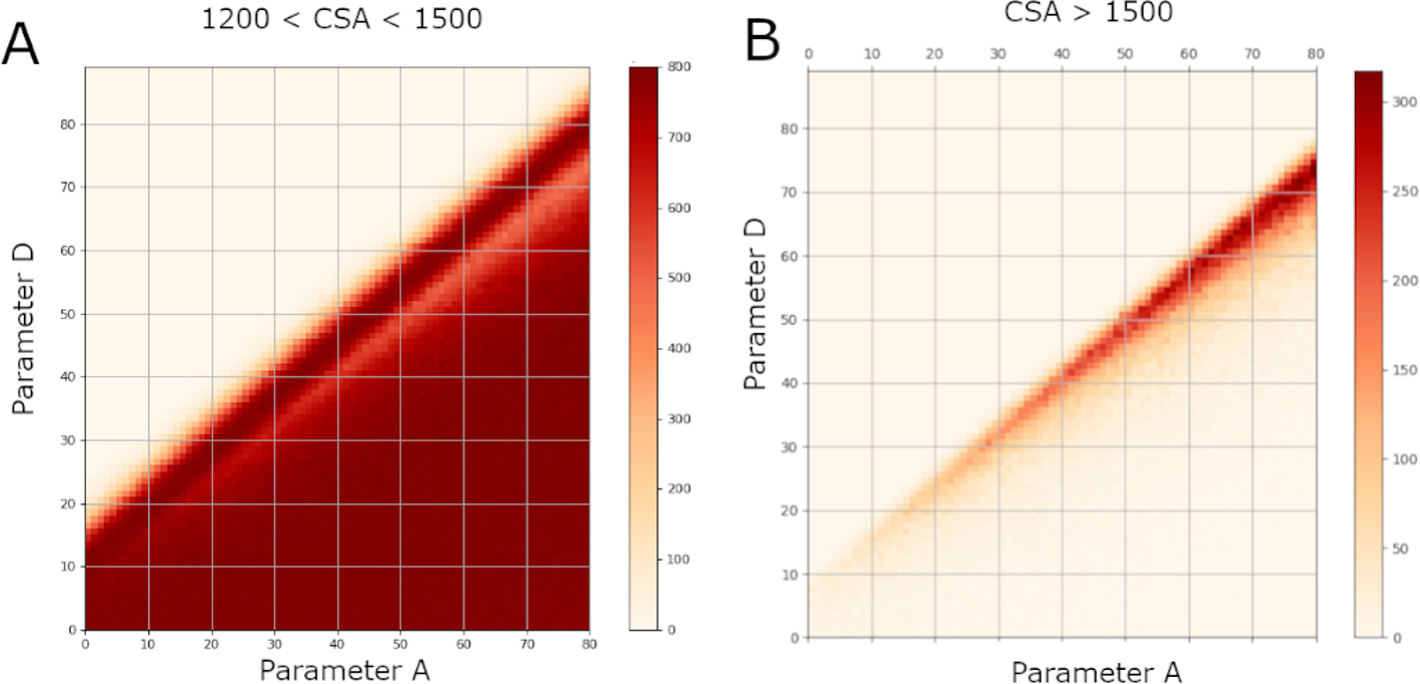
Bidimensional histogram plot for parameter MFavOvrAll-A vs Select-D. (A) shows the frequency of which a combination of MFavOvrAll-A and Select-D achieved a cumulative success area between 1200 and 1500. (B) shows the frequency of which a combination of MFavOvrAll-A and Select-D achieved a cumulative success area greater than 1500.

Figure 11 shows a bidimensional histogram plot for MFavOvrAll-A vs Select-D where Figure 11A is for cumulative success areas between 1200 and 1500 and Figure 11B is for cumulative success areas greater than 1500. On the right graph in Figure 11, we can see a clear relationship between parameters MFavOvrAll-A and Select-D for cumulative success areas between 1200 and 1500. This area can approximately be described by combinations that satisfy the inequality *D* < 0.86*A* + 15.

There is a band missing from Figure 11A that is explained by Figure 11B. Figure 11B represents the highest achieving parameter combinations and appears to require a strict balance between MFavOvrAll-A and Select-D that broadens as the parameter values increase. This relationship between the parameter MFavOvrAll-A and the parameter Select-D can be seen in Figure 10D.

**Figure 12:**
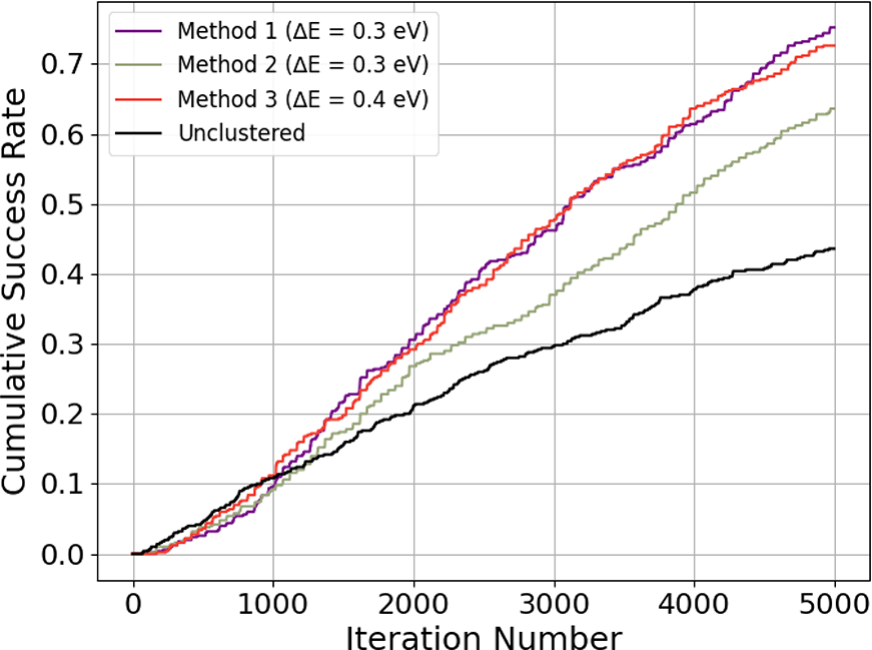
Cumulative success rate of each method with its optimal energy threshold difference Δ*E*. An unclustered EA ran for the same number of iterations is included for comparison.

The cumulative success distribution for the unclustered evolutionary algorithm vs the clustered evolutionary algorithm with the optimal imposed energy threshold, Δ*E*, can be seen in Figure 12. Method 1, Method 2, and Method 3 refer to the different methods we employed to curate the starting population at each iteration (see Section 2.5 for further detail). The unclustered EA consisted of EA runs of 5000 iterations, while the clustered EAs had 100 EA runs consisting of 50 iterations. The cluster-selection EA method used the optimal integer parameters MFavOvrAll-A = 79, MFavClus-B = 3, NoNewFavClus-C = 19, and Select-D = 68 for the dynamic cluster selection for all three methods. By the end of the 5000 iterations, the unclustered EA had a cumulative success rate of 0.436. With a Δ*E* = 0.3, methods 1 and 2 has final cumulative success rates of 0.752 and 0.636, respectively. With Δ*E* = 0.4, method 3 had a final cumulative success rate of 0.726. The unclustered EA begins with a larger cumulative success graph. However, by iteration 1221, all 3 cluster-selection EA methods outperform the unclustered EA method.

**Figure 13:**
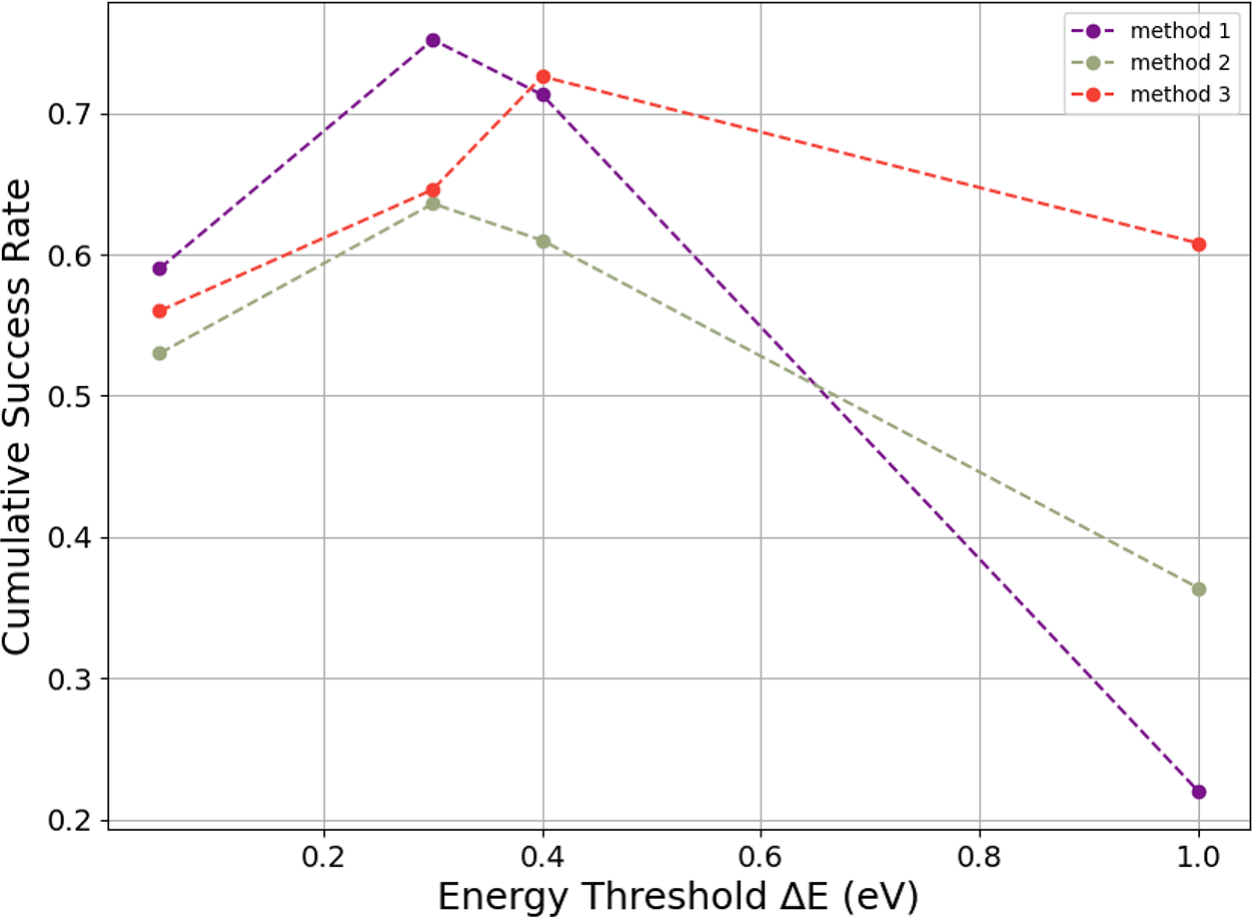
Scatter plot of energy threshold Δ*E* vs cumulative success rate for all three clustering methods.

The cumulative success rates for different energy threshold values, Δ*E*, can be seen in Figure 13. Although method 1 has the highest overall cumulative success rate when Δ*E* = 0.3 eV, the cumulative success drops significantly at higher Δ*E* values. On the contrary, method 3, has a slightly lower best performance (compared to method 1) but has more consistent performance at different Δ*E* values. Method 2 has slightly more stable performance at larger Δ*E* values but under performs both methods 1 and 3 for most energies.

## 4 Discussion

From the Results section, it is clear that we have developed a method for a dynamic cluster selection based evolutionary algorithm that has outperformed a regular unclustered evolutionary algorithm in our search for optimal structures consisting of 9 carbon atoms, 7 hydrogen atoms, and a nitrogen atom.

The steps of our dynamic cluster selection based evolutionary algorithm can be summarized in three steps.

1 Clustering molecules using Oganov et al.’s^34^ fingerprint function, the cosine distance function, and hierarchical agglomerative clustering.
2 Modeling each cluster’s EA output as a Gaussian distribution to optimize learning parameters.
3 Running the cluster-selection evolutionary algorithm with the optimal learning parameters.

Our first step of clustering the molecules follows similar research^11^ with the exception that we’ve replaced the bag of bonds method with Oganov et al.’s fingerprint function. While successful for fingerprinting quinoline-like structures in this paper, we realize that more testing could have been done to find values of δ and Δ that were more sensitive to differences in our structure. This is something that we suspect will especially need to be tuned for larger structures or unit cells. For example, adapting this fingerprint to larger chemical structures may be challenging as small structural differences may be overwhelmed by the size of the structure. While this fingerprint worked well for our molecules, we recognize that a different fingerprinting method may be more suitable for future work with larger structures.

Using DFTB+ as an energy calculator allows us to very quickly estimate a structure’s energy though the accuracy can be questionable at times. For example, we do not believe that 4h-quinolizin-4-ylidene is actually the lowest energy version of C_9_H_7_N. However, the accuracy of this calculator is not a large concern for our method. The reinforcement learning based cluster selection method we employ prioritizes a cluster based on its ability to produce lower and lower energy structures. We could switch DFTB+ with any desired calculator and should expect similar results.

We consider the process of defining a set of learning parameters and tuning them with Gaussian approximations of cluster performance to be where we ventured farthest from previous research. Before deciding to create our own learning parameters, we looked into reinforcement learning solutions to the multiarmed bandit problem such as the epsilon-greedy strategy^36^ and the upper confidence bound (UCB) strategy.^37^ We ultimately decided to create our own set of personalized learning parameters with the hope that it would outperform these generalized reinforcement learning strategies. While we leave the testing of these generalized reinforcement learning methods to future work, we recognize that the lack of notable effect from parameter MFavClus-B and NoNewFavClus-C has left our model looking extremely similar to the upper confidence bound strategy where one term rewards the system for consistently good performance and the second term penalizes systems that are overselected. The notable difference between our method and UCB is that our method rewards a cluster’s ability to create the lowest energy molecule seen so far, whereas UCB rewards average performance.

Similar to other solutions to the multiarmed bandit problem, we found the need for balance between exploration and exploitation to appear organically in our results in two areas. First, we can see the need for this balance by the most optimal learning parameter combinations requiring a strict balance between parameter MFavOvrAll-A (bonus for finding optimal structures) and parameter Select-D (penalty for a cluster being overselected). Parameter MFavOvrAll-A represents exploitation in our search since a cluster that is producing favorable molecules would be repeatedly selected if this parameter were to be set to an extremely large value. On the other hand, molecules would be selected almost randomly if parameter Select-D were to be set to an extremely large value. This would represent a thorough exploration of each cluster regardless of its output.

The second area where we see the need to balance exploration vs exploitation is in the balance between clustering molecules for similarity and imposing energy thresholds to force dissimilarity between molecules. Oganov et al. stated that dissimilar molecules tend to produce unfavorable offspring. However, he does also state that if parent molecules are too similar the EA can stagnate.^17,20^ Our results show that the clustered EA can be boosted by implementing an energy difference threshold between members of the parent population. Therefore, within each cluster, we can conclude that there is an optimal way to sample a parent population that balances picking the lowest energy molecules (exploitation) and including diversity in the parent population (exploration).

We trained our learning parameters with the intention that each evolutionary algorithm could be parallelized to increase the efficiency. During the course of our research, we realized the importance of improving upon the parent population and had changed our method from 20 batch restarts with 5 evolutionary algorithms of 50 iterations running simultaneously to 100 evolutionary algorithms of 50 iterations running serially. We acknowledge that if our Gaussian distribution model used to optimize the learning parameters had been updated for 100 evolutionary algorithms consisting of 50 iterations, then the learning parameters may vary slightly from our current optimum.

The MFavOvrAll-A, MFavClus-B, NoNewFavClus-C, and Select-D parameters are solely based on the ability of a genetic algorithm to keep producing lower energy molecules than those already been generated. We believe that this would allow for a sufficient search for most chemical structures. However, there are considerations that we plan to implement in future work. The learning parameters were not normalized for the number of structures. In other words, MFavOvrAll-A, MFavClus-B, NoNewFavClus-C, and Select-D are specifically set for 9 clusters with initial weights of 100. Systems with a number of clusters notable different from 9 may experience suboptimal performance. Notably larger structures may receive rewards significantly less often and may need an adjustment, as well. In future implementations of this work, we plan to account for the number of cluster normalization. The goal for this implementation would be to allow little to no adjustment of these parameters. In any case, the A, B, C, and D parameters found in this paper could serve as a starting point to any global minimization search following our method. The omission of parameters B and C would also allow for significantly faster optimization.

In order to prevent the parent population of each cluster from becoming filled with duplicates, we imposed varying energy difference requirements on all members of the parent population. For all three methods, the optimal energy difference was found to be the nearest tenth eV. Imposing this threshold had a strong effect on the cumulative success of the method. While further research needs to be done in this area, we believe that the success of the energy threshold in a cluster EA search tells us the ideal parent population requires a balance between similarity and dissimilarity.

In order to impose dissimilarity in the parent population of each cluster, a threshold energy difference, Δ*E*, was implemented. The energy threshold difference was implemented over the fingerprint distance due to its simplicity in both implementation and computational complexity. However, we believe that imposing the fingerprint distance instead of the threshold energy difference would yield similar and possibly better results. One clear limitation of the threshold energy difference is that for larger structures molecules with dissimilar structures may have similar energies. Because all three methods appear to have an ideal energy threshold between 0.2 and 0.5, we suspect this would translate into their existing ideal fingerprint threshold range as well.

Two cases where the effect of clustering was directly related to the efficacy of EA are works by Jørgensen et al. and Hartke.^12,38^ In Jørgensen et al. the EA was clustered and selecting outliers, structures that were outside of existing clusters, were favored to be selected as a parent, while structures from larger clusters were penalized. This resulted in a success rate increase from 28% to 41% in a TiO_2_ edge reconstruction over 2000 iterations. In this case, exploration appears to be prioritized over exploitation, however, it seems to produce favorable results. In the case of Hartke, Lennard-Jones clusters are examined using an EA. Clusters with 75–77 atoms typically take 20–40 times the computational resources as clusters with 74 or 78 atoms. However, by setting up a method that discriminates between different types of atomic clusters and ensuring diversity in the population, the resources required drop to 5–10 times the resources required to find the global minimum structure for clusters with 74 or 78 atoms. Based on these results, this paper is achieving similar speedups; however, our learning agent is balancing both exploration and exploitation. With further study, we predict that the methodology here might outperform these two methods that focus purely on promoting exploration.

## 5 Conclusion

In this paper, we developed a method that could further increase the efficiency of clustering in evolutionary algorithms by optimizing the procedure in which clusters are selected. In this demonstration, we clustered 812 generated quinoline-like molecules into 9 clusters, sampled the output of evolutionary algorithms from each cluster and modeled them as Gaussian distributions, and optimized a set of learning parameters by running simulations with these models. We called these parameters MFavOvrAll-A, MFavClus-B, NoNewFavClus-C, and Select-D where each parameter will increase or decrease the probability of a cluster being selected if its conditions is satisfied.

The highest performing parameter combinations require a fine balance between parameter MFavOvrAll-A (reward for producing the lowest energy molecule in the trial) and parameter Select-D (penalty for appearance) that is emblematic of the need to balance exploration vs exploitation in evolutionary algorithm-based searches and general reinforcement learning-based solutions.

In order to prevent stagnation within the evolutionary algorithm, we required a minimum Δ*E* between members of the parent population. We believe that the success of this Δ*E* implementation demonstrates the need to prevent premature convergence that can be accelerated by clustering parent populations. In doing so, our parents become similar enough to produce more favorable offspring but dissimilar enough to avoid converging on a local minima (funneling).

Taking the highest performing parameter combination, we were able to achieve a cumulative success rate of 0.752 after 5000 iterations. We found this to be a notable increase compared to the cumulative success rate of 0.436 yielded by the unclustered EA after 5000 iterations.

While future work needs to be done to generalize this method, the results of this paper show that reinforcement learning based methods of cluster selection are successful in increasing the efficiency of evolutionary algorithm searches for global minima molecular structures. We plan to further improve this method so that it can be used for future EA searches to determine the morphology of the physical defects in materials.

## References

[ref1] Goedecker S. Minima hopping: An efficient search method for the global minimum of the potential energy surface of complex molecular systems. J. Chem. Phys. 2004, 120, 9911–9917. 10.1063/1.1724816. 15268009

[ref2] Wales D. J.; Doye J. P. K. Global Optimization by Basin-Hopping and the Lowest Energy Structures of Lennard-Jones Clusters Containing up to 110 Atoms. J. Phys. Chem. A 1997, 101, 5111–5116. 10.1021/jp970984n.

[ref3] Rondina G. G.; Da Silva J. L. F. Revised Basin-Hopping Monte Carlo Algorithm for Structure Optimization of Clusters and Nanoparticles. J. Chem. Inf. Model. 2013, 53, 2282–2298. 10.1021/ci400224z. 23957311

[ref4] Pickard C. J.; Needs R. J. Ab initio random structure searching. J. Phys.: Condens. Matter 2011, 23, 053201 10.1088/0953-8984/23/5/053201. 21406903

[ref5] Jana G.; Mitra A.; Pan S.; Sural S.; Chattaraj P. Modified Particle Swarm Optimization Algorithms for the Generation of Stable Structures of Carbon Clusters, C n (n = 3–6, 10). Frontiers in Chemistry 2019, 10.3389/fchem.2019.00485. PMC6640203 31355182

[ref6] Wang Y.; Lv J.; Zhu L.; Ma Y. Crystal structure prediction via particle-swarm optimization. Phys. Rev. B 2010, 82, 094116 10.1103/PhysRevB.82.094116.

[ref7] Kirkpatrick S. Jr; Gelatt C.; Vecchi M. Optimization by simulated annealing. Science 1983, 220, 671–680. 10.1126/science.220.4598.671. 17813860

[ref8] Zhai H.; Alexandrova A. N. Ensemble-Average Representation of Pt Clusters in Conditions of Catalysis Accessed through GPU Accelerated Deep Neural Network Fitting Global Optimization. J. Chem. Theory Comput. 2016, 12, 6213–6226. 10.1021/acs.jctc.6b00994. 27951667

[ref9] Deaven D. M.; Ho K. M. Molecular Geometry Optimization with a Genetic Algorithm. Phys. Rev. Lett. 1995, 75, 288–291. 10.1103/PhysRevLett.75.288. 10059656

[ref10] Vilhelmsen L.; Hammer B. A genetic algorithm for first principles global structure optimization of supported nano structures. J. Chem. Phys. 2014, 141, 044711 10.1063/1.4886337. 25084941

[ref11] Kellas N.; Groves M. Improvement of the Evolutionary Algorithm on the Atomic Simulation Environment Though Intuitive Starting Population Creation and Clustering. Journal of Computational Science Education 2020, 11, 29–35. 10.22369/issn.2153-4136/11/2/5.

[ref12] Jørgensen M. S.; Groves M. N.; Hammer B. Combining Evolutionary Algorithms with Clustering toward Rational Global Structure Optimization at the Atomic Scale. J. Chem. Theory Comput. 2017, 13, 1486–1493. 10.1021/acs.jctc.6b01119. 28186745

[ref13] Silva F. T.; Silva M. X.; Belchior J. C. A New Genetic Algorithm Approach Applied to Atomic and Molecular Cluster Studies. Frontiers in Chemistry 2019, 10.3389/fchem.2019.00707. PMC6848380 31750290

[ref14] Alexandrova A. N. H· (H2O)n Clusters: Microsolvation of the Hydrogen Atom via Molecular ab Initio Gradient Embedded Genetic Algorithm (GEGA). J. Phys. Chem. A 2010, 114, 12591–12599. 10.1021/jp1092543. 21077611

[ref15] Curtis F.; Li X.; Rose T.; Vázquez-Mayagoitia Á.; Bhattacharya S.; Ghiringhelli L. M.; Marom N. GAtor: A first-principles genetic algorithm for molecular crystal structure prediction. J. Chem. Theory Comput. 2018, 14, 2246–2264. 10.1021/acs.jctc.7b01152. 29481740

[ref16] Morris J. R.; Deaven D. M.; Ho K. M. Genetic-algorithm energy minimization for point charges on a sphere. Phys. Rev. B 1996, 53, R1740–R1743. 10.1103/PhysRevB.53.R1740. 9983695

[ref17] Lyakhov A. O.; Oganov A. R.; Valle M. How to predict very large and complex crystal structures. Comput. Phys. Commun. 2010, 181, 1623–1632. 10.1016/j.cpc.2010.06.007.

[ref18] Jørgensen M. S.; Larsen U. F.; Jacobsen K. W.; Hammer B. Exploration versus Exploitation in Global Atomistic Structure Optimization. J. Phys. Chem. A 2018, 122, 1504–1509. 10.1021/acs.jpca.8b00160. 29314842

[ref19] Pereira F.; Marques J. A study on diversity for cluster geometry optimization. Evolutionary Intelligence 2009, 2, 121–140. 10.1007/s12065-009-0020-5.

[ref20] Curtis F.; Rose T.; Marom N. Evolutionary niching in the GAtor genetic algorithm for molecular crystal structure prediction. Faraday Discuss. 2018, 211, 61–77. 10.1039/C8FD00067K. 30073236

[ref21] Loeffler T. D.; Chan H.; Gray S.; Sankaranarayanan S. K. R. S. Teamwork Makes the Dream Work”: Tribal Competition Evolutionary Search as a Surrogate for Free-Energy-Based Structural Predictions. J. Phys. Chem. A 2019, 123, 3903–3910. 10.1021/acs.jpca.9b00914. 30939871

[ref22] Bahn S.; Jacobsen K. An object-oriented scripting interface to a legacy electronic structure code. Computing in Science & Engineering 2002, 4, 56–66. 10.1109/5992.998641.

[ref23] Hjorth Larsen A.; et al. The Atomic Simulation Environment — A Python library for working with atoms. J. Phys.: Condens. Matter 2017, 29, 273002 10.1088/1361-648X/aa680e. 28323250

[ref24] Aradi B.; Hourahine B.; Frauenheim T. DFTB+, a Sparse Matrix-Based Implementation of the DFTB Method. J. Phys. Chem. A 2007, 111, 5678–5684. 10.1021/jp070186p. 17567110

[ref25] Frenzel J.; Oliveira A.; Jardillier N.; Heine T.; Seifert G. Semi-relativistic, self-consistent charge Slater-Koster tables for density-functional based tight-binding (DFTB) for materials science simulations. 2009. http://www.dftb.org/parameters/download/matsci/ (accessed 2024-03-11).

[ref26] Lukose B.; Kuc A.; Frenzel J.; Heine T. On the reticular construction concept of covalent organic frameworks. Beilstein journal of nanotechnology 2010, 1, 60–70. 10.3762/bjnano.1.8. 21977395 PMC3045923

[ref27] Tom R.; Rose T.; Bier I.; O’Brien H.; Vázquez-Mayagoitia Á.; Marom N. Genarris 2.0: A random structure generator for molecular crystals. Comput. Phys. Commun. 2020, 250, 107170 10.1016/j.cpc.2020.107170.

[ref28] Carhart R. E.; Smith D. H.; Venkataraghavan R. Atom pairs as molecular features in structure-activity studies: definition and applications. J. Chem. Inf. Comput. Sci. 1985, 25, 64–73. 10.1021/ci00046a002.

[ref29] Hansen K.; Biegler F.; Ramakrishnan R.; Pronobis W.; von Lilienfeld A.; Müller K.-R.; Tkatchenko A. Machine Learning Predictions of Molecular Properties: Accurate Many-Body Potentials and Non-Locality in Chemical Space. J. Phys. Chem. Lett. 2015, 6, 2326–2331. 10.1021/acs.jpclett.5b00831. 26113956 PMC4476293

[ref30] Behler J.; Parrinello M. Generalized Neural-Network Representation of High-Dimensional Potential-Energy Surfaces. Phys. Rev. Lett. 2007, 98, 146401 10.1103/PhysRevLett.98.146401. 17501293

[ref31] Bartók A. P.; Kondor R.; Csányi G. On representing chemical environments. Phys. Rev. B 2013, 87, 184115 10.1103/PhysRevB.87.184115.

[ref32] Parsaeifard B.; Goedecker S. Manifolds of quasi-constant SOAP and ACSF fingerprints and the resulting failure to machine learn four-body interactions. J. Chem. Phys. 2022, 156, 034302 10.1063/5.0070488. 35065570

[ref33] Schütt K. T.; Glawe H.; Brockherde F.; Sanna A.; Müller K. R.; Gross E. K. U. How to represent crystal structures for machine learning: Towards fast prediction of electronic properties. Phys. Rev. B 2014, 89, 205118 10.1103/PhysRevB.89.205118.

[ref34] Valle M.; Oganov A. Crystal fingerprint space - A novel paradigm for studying crystal-structure sets. Acta crystallographica. Section A, Foundations of crystallography 2010, 66, 507–17. 10.1107/S0108767310026395. 20720316

[ref35] Wolf M. D.; Landman U. Genetic Algorithms for Structural Cluster Optimization. J. Phys. Chem. A 1998, 102, 6129–6137. 10.1021/jp9814597.

[ref36] Sutton R. S.; Barto A. G. Reinforcement learning: An introduction; MIT Press: 2018.

[ref37] Auer P. Using confidence bounds for exploitation-exploration trade-offs. Journal of Machine Learning Research 2002, 3, 397–422.

[ref38] Hartke B. Global cluster geometry optimization by a phenotype algorithm with Niches: Location of elusive minima, and low-order scaling with cluster size. J. Comput. Chem. 1999, 20, 1752 10.1002/(SICI)1096-987X(199912)20:16<1752::AID-JCC7>3.0.CO;2-0.

